# Predictive value of noninducibility after catheter ablation for paroxysmal and persistent atrial fibrillation

**DOI:** 10.1002/joa3.12320

**Published:** 2020-03-02

**Authors:** Shinichi Tachibana, Akira Mizukami, Shunsuke Kuroda, Tatsuya Hayashi, Akihiko Matsumura, Masahiko Goya, Tetsuo Sasano

**Affiliations:** ^1^ Department of Cardiology Yokohama City Minato Red Cross Hospital Yokohama Japan; ^2^ Department of Cardiology Kameda Medical Center Kamogawa Japan; ^3^ Cardiovascular Medicine Department Cleveland Clinic Cleveland OH USA; ^4^ Cardiovascular Medicine Tokyo Medical and Dental University Tokyo Japan

**Keywords:** atrial fibrillation, catheter ablation, inducibility, pacing interval, rapid atrial pacing

## Abstract

**Background:**

It is unclear whether pacing maneuver at the end of catheter ablation for atrial fibrillation (AF) predicts recurrence of atrial tachyarrhythmia postintervention.

**Objective:**

To investigate whether the predictive value of incremental pacing maneuver after catheter ablation for AF depends on the pacing cycle length and type of AF.

**Methods:**

This study included 298 consecutive patients who underwent initial catheter ablation for nonvalvular AF (61% paroxysmal AF [PAF], 39% persistent AF [PeAF]). Rapid atrial pacing was performed at the end of the procedure. We analyzed minimum coupling interval (CI) of pacing, arrhythmia‐inducibility, and atrial tachyarrhythmia recurrence in patients with PAF and PeAF.

**Results:**

Patients were divided into the following three groups according to their response to pacing maneuver: AF‐inducible (inducible group; n = 86), noninducible at CI ≥200 ms (non‐CI ≥200 group; n = 100), and noninducible at CI <200 ms (non‐CI <200 group; n = 112). Kaplan‐Meier analysis showed that response to pacing maneuver was significantly associated with recurrence of atrial tachyarrhythmias (*P* = .028). Cox‐regression analysis showed that non‐CI <200 was an independent predictor when the inducible group was used as a reference (hazard ratio 0.60, 95% confidence interval 0.40‐0.96, *P* = .031). However, when PAF and PeAF were analyzed separately, non‐CI <200 was an independent predictor only in PeAF.

**Conclusion:**

Noninducibility with shorter CI predicted atrial tachyarrhythmia recurrence only for PeAF. Pacing CI and type of AF could influence the predictive value of atrial tachyarrhythmia recurrence.

## Graphical abstract

When patients were divided into three groups according to the response to pacing maneuver (inducible, non‐inducible at coupling interval [CI] ≥ 200 ms, and non‐inducible at CI < 200 ms), significant difference in atrial tachyarrhythmia free survival were observed (panel A). However, when paroxysmal atrial fibrillation (PAF) and persistent atrial fibrillation (PeAF) were analyzed separately, significant deference was observed only in PeAF (panel B and C).

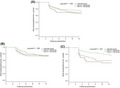

## 1 INTRODUCTION

Pulmonary vein isolation (PVI) is an effective treatment for atrial fibrillation (AF) and has been performed in many hospitals in recent years. The prognostic value of noninducibility of AF with atrial burst pacing at the end of procedure has been evaluated in prior studies.^1^, ^2^, ^3^, ^4^, ^5^, ^6^, ^7^, ^8^, ^9^, ^10^ However, the results of these previous studies were variable, and the predictive values of AF inducibility obtained so far in studies are controversial. This may be due to the differences in induction protocols, definitions of inducibility, and type of AF among previous studies. Little is known about the difference in inducibility due to rapid atrial pacing (RAP) between paroxysmal AF (PAF) and persistent AF (PeAF). Moreover, no study has examined the relationship between atrial tachyarrhythmia recurrence rate and noninducibility with varying atrial pacing cycle lengths after PVI. We aimed to investigate whether the predictive value of the incremental pacing maneuver after catheter ablation depends on the pacing cycle length and type of AF.

## 2 METHODS

### 2.1 Study population

The subjects were consecutive patients undergoing AF ablation for standard clinical indications at Kameda Medical Center.^11^, ^12^ We provided written informed consent for ablation procedures for all patients. Exclusion criteria were as follows: additional ablation for AF was performed after incremental pacing and prior history of catheter ablation.

Definitions of PAF and PeAF were according to the American Heart Association/American College of Cardiology/European Society of Cardiology guidelines,^13^ which are as follows: PAF was defined as AF that self‐terminated within 7 days, whereas PeAF was defined as continuous AF lasting for more than 7 days.

### 2.2 Catheter ablation procedure

All patients received anticoagulation therapy for ˃3 weeks before catheter ablation. Antiarrhythmic drugs were discontinued for at least 5 half‐lives before the procedure, and none of the patients received amiodarone before the procedure.

Catheter ablation was performed under deep sedation using fentanyl, propofol, and dexmedetomidine. Vascular access was acquired through the subclavian and femoral veins. A 20 pole catheter (BeeAT, Japan Lifeline) was inserted in the coronary sinus for electrogram recording, atrial pacing, and defibrillation. Following septal puncture, anticoagulation was achieved with intravenous heparin, and activated clotting time of >300 seconds was maintained. During catheter ablation, CARTO system (Biosense Webster) or EnSite NavX navigation system (St. Jude Medical) was used for three dimensional mapping.

A catheter (Lasso or Pentaray, Biosense Webster, Optima or Afocus, St. Jude Medical) was used to map the left atrium (LA) and each pulmonary vein (PV). Bipolar electrogram filter was set between 30 and 500 Hz. Radiofrequency energy was set at the maximum output of 40 W, and catheter tip temperature was not allowed to exceed 42°C. When the esophageal temperature reached >39°C, no more energy was delivered.

When a cryoballoon was used, a 15 Fr sheath (FlexCath Advance) was inserted into the LA and an inner lumen mapping catheter (Achieve, Medtronic, Inc) was preceded in PV. Then, a 28 mm cryoballoon (Arctic Front Advance) was inflated and frozen at each PV ostium. PVI was confirmed with the Achieve catheter. Linear ablation, complex fractionated atrial electrogram (CFAE) ablation, superior vena cava (SVC) isolation, and cavotricuspid isthmus ablation were performed after PVI based on the electrophysiological findings and at the discretion of the operator.

### 2.3 Study protocol

We performed the atrial pacing protocol from the coronary sinus at first. When LA could not be captured by pacing from coronary sinus, stimulation from the right atrium was performed. RAP was performed at the end of catheter ablation (10 beat drive train), from 300 ms with a subsequent decrement of 10 ms or failure to capture the 1:1 atrial tissue. If additional ablation was performed after RAP, RAP was repeated after the additional ablation, and the result of the last RAP was used for analysis. Therefore, no ablation was performed after the analyzed RAP. We defined inducibility as atrial arrhythmia (AF or organized AT) persisting for >1 minute. No isoproterenol was used during the RAP.

### 2.4 Follow‐up

All patients visited the hospital at 1 and 3 months and every 2‐3 months after discharge. On the day of the outpatient visit, patients were asked about the symptoms and 12 lead ECG was recorded. Whenever symptoms considered to be atrial arrhythmia appeared, patients were encouraged to visit the hospital and 12 lead ECG was recorded. An external loop recorder was used for 1 week at 3 months after the procedure. Atrial tachyarrhythmia recurrence was defined as sustained AF/AT lasting for ˃30 seconds, which appeared ˃3 months after the catheter ablation.

### 2.5 Statistical analysis

Continuous variables are expressed as mean ± SD, and significant differences were analyzed by one‐way analysis of variance. Categorical data are expressed as number and percentages, and were compared using *χ* ^2^ test, when appropriate. Univariate and multivariate Cox proportional hazards regression analyses were performed on candidate variables to predict the trichotomous outcome. Variables with *P* < .1 in the univariate analysis were entered into the multivariate analysis. Survival curves were calculated using the Kaplan–Meier method and compared using the Log Rank test. A *P* < .05 was deemed significant. Statistical analyses were conducted using the R software.

## 3 RESULTS

A total of 298 patients (70% male, age 65 ± 9 years) were enrolled in the study. Overall, 182 patients (61%) had PAF and 116 (39%) had PeAF. Mean CHADS2 score was 1.2 ± 1.0 and mean LA diameter was 43 ± 7 mm.

**Figure 1:**
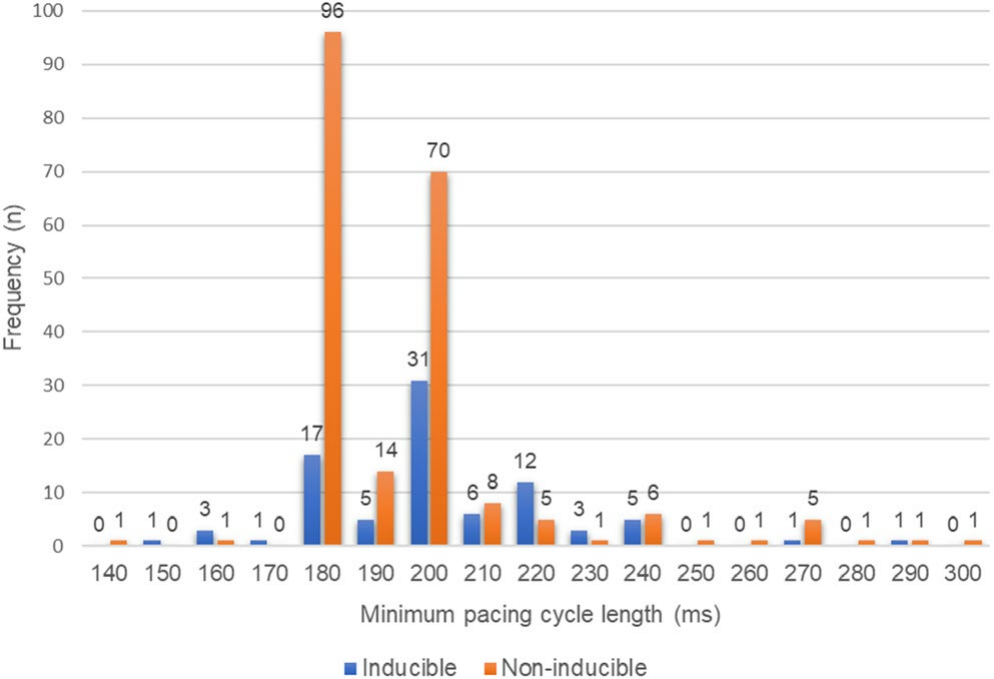
Minimum atrial pacing cycle length and AF/AT inducibility

Pulmonary vein isolation was performed successfully in all patients. At the end of the catheter ablation, sustained atrial tachyarrhythmia could be induced in 86 patients (inducible group; n = 86). Median minimal RAP coupling interval (CI) was 190 ms among noninducible group (Figure 1). With this value, the noninducible patients were divided in half for analysis: patients with CI equal to or longer than 200 ms (non‐CI ≥200 group; n = 100) and those with CI shorter than 200 ms (non‐CI <200 group; n = 112).

Rapid atrial pacing was performed from coronary sinus in 281 patients (94.3%), and right atrium in 17 patients (5.7%). RAP was repeated in 153 patients (51.3%). When AF or organized AT was induced even once, patients were classified into the inducible group. Only 10 patients (6.4%) had different inducibility with the same pacing cycle length at repeated RAP.

Table 1 shows the baseline clinical characteristics of the study patients. There were no significant differences in body mass index (BMI) or comorbidity, such as diabetes and history of congestive heart failure or stroke, among the three groups. AF type (PAF or PeAF), LA diameter, and left ventricular ejection fraction (LVEF) were also comparable among the three groups. The non‐CI <200 group was younger (*P* = .002), had more male patients (*P* = .002), was less likely to have hypertension (*P* = .014), and had lower CHADS2 scores (*P* = .038).

**Table 1:** Baseline patient characteristics

Variable	Overall (n = 298)	Inducible (n = 86)	Noninducible (n = 212)		*P* value*, *
			Non‐CI ≥200 (n = 100)	Non‐CI <200 (n = 112)	
Age, y	65 ± 9	66 ± 8	66 ± 9	62 ± 9	.002
Male, n	208 (70.0%)	50 (58.1%)	67 (67.0%)	91 (81.3%)	.002
PAF, n	182 (61.1%)	53 (61.6%)	63 (63.0%)	66 (58.9%)	.839
PeAF, n	116 (38.9%)	33 (38.4%)	37 (37.0%)	46 (41.1%)	.839
BMI, kg/m^2^	25 ± 4	25 ± 4	24 ± 4	25 ± 4	.37
Hypertension, n	163 (54.7%)	56 (65.1%)	57 (57.0%)	50 (44.6%)	.014
Diabetes, n	52 (17.4%)	13 (15.1%)	13 (13.0%)	26 (23.2%)	.117
Heart failure, n	45 (15.1%)	16 (18.6%)	16 (16.0%)	13 (10.8%)	.377
Prior stroke/TIA, n	27 (9.1%)	12 (14.0%)	8 (8.0%)	7 (6.3%)	.157
CHADS2 score					.038
0	85 (28.5%)	22 (25.6%)	26 (26.0%)	37 (33.0%)	
1	116 (38.9%)	32 (37.2%)	41 (41.0%)	43 (38.4%)	
≥2	97 (32.6%)	32 (37.2%)	33 (33.0%)	32 (28.6%)	
LA diameter, mm	43 ± 7	43 ± 7	43 ± 7	42 ± 6	.777
LVEF, %	65 ± 10	65 ± 11	65 ± 10	65 ± 9	.824

Note

Values are expressed as mean ± SD or as n (%).

Abbreviations: AF, atrial fibrillation; BMI, body mass index; CHADS2 score, congestive heart failure, age ˃75 y, diabetes mellitus, previous stroke/transit ischemic attack; LA, left atrium; LVEF, left ventricular ejection fraction; PAF, paroxysmal atrial fibrillation; PeAF, persistent atrial fibrillation; TIA, transient ischemic attack.

* *P* value is compared between the inducible, non‐CI ≥200, and non‐CI <200 groups, respectively.

Table 2 summarizes the characteristics of the catheter ablation method. Cryoballoon ablation and SVC isolation were less frequent in the non‐CI <200 group.

**Table 2:** Characteristics of catheter ablation

Variable	Overall (n = 298)	Inducible (n = 86)	Noninducible (n = 212)		*P* value*
			Non‐CI ≥200 (n = 100)	Non‐CI <200 (n = 112)	
Cryoballoon ablation, n	37 (12.4%)	14 (16.3%)	17 (17.0%)	6 (5.4%)	.012
Posterior isolation, n	19 (6.4%)	8 (9.3%)	7 (7.0%)	4 (3.6%)	.258
Roof line ablation, n	23 (7.7%)	7 (8.1%)	7 (7.0%)	9 (8.0%)	.927
CFAE, n	10 (3.4%)	5 (5.8%)	2 (2.0%)	3 (2.7%)	.338
MI line ablation, n	8 (2.7%)	5 (5.8%)	2 (2.0%)	1 (0.9%)	.113
SVC isolation, n	17 (5.7%)	8 (9.3%)	7 (7.0%)	2 (1.8%)	.039
CTI ablation, n	145 (48.7%)	43 (50.0%)	52 (52.0%)	60 (53.6%)	.895

Note

Values are expressed as mean ± SD or as n (%).

Abbreviation: CFAE, complex fractionated atrial electrogram; CTI, cavotricuspid isthmus; MI, mitral isthmus; SVC, superior vena cava.

* *P* value obtained when comparing the inducible, non‐CI ≥200, and non‐CI <200 groups.

Among inducible group, organized AT was induced in eight patients (9.3%). There were no significant differences in baseline characteristics and catheter ablation methods between AF inducible and organized AT inducible groups.

### 3.1 Follow‐up

**Figure 2:**
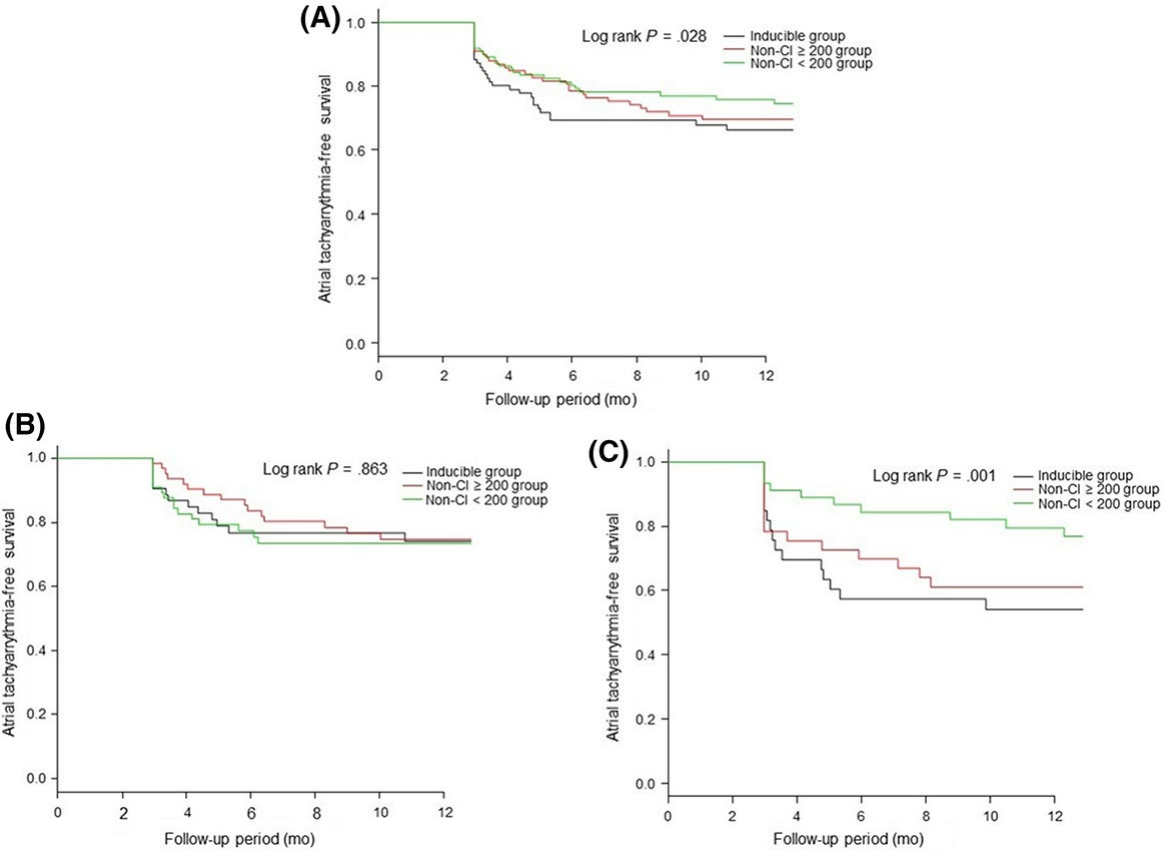
Long‐term freedom from atrial tachyarrhythmia recurrence according to the result of inducibility status at the end of catheter ablation in all patients (A), in patients with PAF (B), and in patients with PeAF (C)

After a mean follow‐up of 29 ± 21 months, cumulative atrial tachyarrhythmia free survival was 56% of patients in the inducible group, 62% of patients in the non‐CI ≥200 group, and 71% of patients in the non‐CI <200 group. There was a significant difference in atrial tachyarrhythmia‐free survival among the three groups (Log‐rank *P* = .028; Figure 2). In the Cox regression analysis, the non‐CI <200 group was a significant independent predictive factor of atrial tachyarrhythmia recurrence (hazards ratio [HR] = 0.60, 95% confidence interval [CI] 0.40‐0.96, *P* = .031; Table 3).

**Table 3:** Predictor of atrial tachyarrhythmia recurrence after catheter ablation

	Univariate analysis		*P*‐value	Multivariate analysis		*P*‐value
	HR	(95% CI)		HR	(95% CI)	
Age, y	0.99	0.97‐1.02	.59			
Male	0.8	0.54‐1.19	.27			
LVEF	0.99	0.97‐1.01	.22			
BMI, kg/m^2^	0.99	0.94‐1.04	.59			
LA diameter	1.03	1.00‐1.06	.024	1.02	0.99‐1.05	.17
PeAF	1.46	1.00‐2.13	.049	1.24	0.83‐1.86	.28
AAD use during follow‐up	1.88	1.27‐2.78	.002	1.58	1.05‐2.40	.029
Compared with the inducible group						
Non‐CI ≥200	0.78	0.49‐1.22	.27			
Non‐CI <200	0.53	0.33‐0.86	.009	0.6	0.40‐0.96	.031

Abbreviations: AAD, antiarrhythmic drugs; BMI, body mass index; CI, confidence interval; HR, hazard ratio; LA, left atrium; LVEF, left ventricular ejection fraction; PeAF, persistent atrial fibrillation.

### 3.2 Type of AF and inducibility

Kaplan–Meier analysis shows that, in patients with PAF, there was no difference in recurrence among the three groups (Log‐rank *P* = .863). However, in patients with PeAF, there was a significant difference in recurrence among the three groups (Log‐rank *P* = .001; Figure 2).

Cox regression analysis showed that the non‐CI <200 group was not an independent predictive factor of atrial tachyarrhythmia recurrence in patients with PAF (hazards ratio [HR] = 0.89, 95% confidence interval [CI] 0.46‐1.72, *P* = .73; Table 4). However, the non‐CI <200 group was a significant independent predictive factor of atrial tachyarrhythmia recurrence in patients with PeAF (hazards ratio [HR] = 0.41, 95% confidence interval [CI] 0.21‐0.78, *P* < .01; Table 5).

**Table 4:** Predictor of atrial tachyarrhythmia recurrence after catheter ablation in patients with PAF

	Univariate analysis		*P*‐value	Multivariate analysis		*P*‐value
	HR	(95% CI)		HR	(95% CI)	
Age, years	1.0	0.97‐1.03	.79			
Male	0.96	0.55‐1.68	.88			
LVEF	0.99	0.96‐1.03	.65			
BMI, kg/m^2^	0.97	0.9‐1.04	.38			
LA diameter	1.0	0.96‐1.04	.98			
AAD use during follow‐up	1.94	1.04‐3.63	.04	1.94	1.04‐3.63	.04
Compared with the inducible group						
Non‐CI ≥200	0.83	0.43‐1.62	.59			
Non‐CI <200	0.89	0.46‐1.72	.73			

Abbreviations: AAD, antiarrhythmic drugs; BMI, body mass index; CI, confidence interval; HR, hazard ratio; LA, left atrium; LVEF, left ventricular ejection fraction; PAF, paroxysmal atrial fibrillation.

**Table 5:** Predictor of atrial tachyarrhythmia recurrence after catheter ablation in patients with PeAF

	Univariate analysis		*P*‐value	Multivariate analysis		*P*‐value
	HR	(95% CI)		HR	(95% CI)	
Age, y	0.99	0.96‐1.03	.75			
Male	0.58	0.33‐1.03	.063	0.69	0.39‐1.23	.2
LVEF	1.0	0.97‐1.02	.72			
BMI, kg/m^2^	0.98	0.91‐1.05	.59			
LA diameter	1.05	1.01‐1.1	.014	1.05	1.01‐1.09	.03
AAD use during follow‐up	1.62	0.95‐2.78	.076	1.48	0.86‐2.56	.16
Compared with the Inducible group						
Non‐CI ≥200	0.78	0.39‐1.35	.32			
Non‐CI <200	0.29	0.15‐0.6	<.01	0.41	0.21‐0.78	<.01

Abbreviations: AAD, antiarrhythmic drugs; BMI, body mass index; CI, confidence interval; HR, hazard ratio; LA, left atrium; LVEF, left ventricular ejection fraction; PeAF, persistent atrial fibrillation.

### 3.3 Induced and recurrent atrial arrhythmias

There appears to be no apparent relationship between induced type and recurrence type of atrial tachyarrhythmia. Of the 13 patients who had recurrence with AT during follow‐up, only three patients had inducible organized AT after catheter ablation. Of the eight patients who had organized AT induction after catheter ablation, two patients had recurrence of AF and only one patient had recurrence of AT during follow‐up.

### 3.4 Antiarrhythmic drugs after catheter ablation

Antiarrhythmic drugs were used in 75/298 (25.2%) of the patients during follow‐up, and the rate was significantly different between inducible group, non‐CI ≥200 group, and non‐CI <200 group (33 [38.4%], 19 [19.0%], and 23 [20.5%] patients respectively, *P* < .01). The non‐CI <200 remained to be an independent predictor of recurrence after adjustment with antiarrhythmic drug use during follow‐up in the whole cohort and in patients with PeAF (Tables 3 and 5). In this study, antiarrhythmic drug use during follow‐up increased the risk of recurrence. This is a retrospective analysis, and AAD may have been prescribed to patients with higher risk of recurrence.

### 3.5 Predictive values of atrial burst pacing

Sensitivity and specificity as well as positive and negative predictive values of the response to pacing maneuver for prediction of atrial tachyarrhythmia recurrence at 6 months are shown in Table 6.

**Table 6:** Accuracy of rapid atrial pacing to predict atrial tachyarrhythmia recurrence 6 mo after procedure

	Sensitivity (%)	Specificity (%)	PPV (%)	NPV (%)
Inducible (vs non‐CI ≥200 + non‐CI <200)	40.0%	74.2%	30.2%	81.6%
Inducible + non‐CI ≥200 (vs non‐CI <200)	69.2%	39.5%	24.2%	82.1%

Abbreviations: NPV, negative predictive value; PPV, positive predictive value.

## 4 DISCUSSION

In the present retrospective study, we analyzed 298 patients who underwent initial catheter ablation for AF and evaluated whether the predictive value of RAP depends on the pacing cycle length. The main finding of our study was as follow: noninducibility with short RAP cycle length has significant predictor of improved atrial tachyarrhythmia‐free survival during follow‐up. However, when PAF and PeAF were assessed separately, this finding was significant only in the PeAF group.

### 4.1 Inducibility by RAP

The prognostic value of noninducibility of atrial tachyarrhythmia with RAP at the end of catheter ablation has been investigated in several prior observational studies. However, induction methods and definitions of atrial tachyarrhythmia inducibility differed among previous studies^1^, ^2^, ^3^, ^4^, ^5^, ^6^, ^7^, ^8^, ^9^, ^10^ (Table 7). Some authors argued that noninducibility of AF with RAP at the end of procedure is suitable for assessing the likelihood of recurrence.^1^, ^2^, ^3^ Other authors decided to add linear ablation using AF inducibility as an indicator.^4^, ^5^, ^14^ Noninducibility has been regarded as an endpoint of catheter ablation modifying the substrate of AF.^15^, ^16^, ^17^

**Table 7:** Summary of prior studies evaluating atrial tachyarrhythmia noninducibility as endpoint of catheter ablation

Author & study year	Sample size	AF type	Induction protocol	Definition of inducibility	Follow‐up month	AF‐ or AT‐free survival of the Noninducible group	AF‐ or AT‐free survival of the Inducible group	*P*‐value*, *
Haissaguerre et al (2004)^1^	70	PAF	Decremental burst pacing (5 sec)	AF ≥1 min	7 ± 3	87%	62%	.03
Oral et al (2004)^2^	100	PAF	Burst pacing (≥ 15 sec at shortest cycle length with 1:1 atrial capture)	AF >1 min	6	85%	67%	.05
Essebag et al (2005)^3^	102	PAF (59%), PeAF (41%)	Burst pacing (5 sec at 200 ms)	AF or AT >10 s	12	72%	53%	.04
Jais et al (2006)^8^	74	PAF	Decremental burst pacing (10 sec)	AF >10 min	18 ± 4	91%	NR	
Richter et al (2006)^10^	234	PAF (71%), PeAF (29%)	Decremental burst pacing	AF ≥1 min	6	NR	NR	
Chang et al (2007)^9^	88	PAF	Decremental burst pacing (5‐10 sec)	AF or AT >1 min	12 ± 6	81%	45%	.02
Satomi et al (2008)^11^	60	PAF	Decremental burst pacing (10 sec)	AF >10 min	16 ± 8	58%	59%	
Crawford et al (2010)^12^	112	PAF	Decremental burst pacing (10 sec)	AF ≥1 min	12 ± 5	84%	76%	.03
Leong‐Sit et al (2013)^13^	144	PAF (54%), PeAF (46%)	Decremental burst pacing (15 beats)	AF or AT ≥2 min	12	51%	51%	.65
Santageli et al (2018)^14^	305	PAF (66%), PeAF (34%)	Decremental burst pacing (15 beats)	AF or AT ≥2 min	19 ± 7	76%	69%	.24
Current study	182	PAF	Decremental burst pacing (10 beats)	AF or AT >1 min	29 ± 21	71%	68%	.86
	116	PeAF				60%	36%	.01

Abbreviations: AF, atrial fibrillation; AT, atrial tachycardia; PAF, paroxysmal atrial fibrillation; PeAF, persistent atrial fibrillation.

* *P* value obtained when comparing the inducible AF/AT group with the noninducible AF/AT group.

Previous studies evaluated inductivity mainly for PAF cases. Recent study by Kawai et al reported that AF/AT inducibility could not predict atrial tachyarrhythmia recurrence in patients with PeAF. However, in selected patients with LA diameter of 45 mm or more, the inducibility predicted recurrence.^18^ In our study, mean LA diameter in PeAF patients was 45.1 ± 6.8 mm, which was larger than the previous study, which might have affected the results. Crawford et al reported that PAC in response to isoproterenol predicts AF recurrence more accurately than AF inducibility with RAP after the procedure.^8^ Velagic et al reported that, in PAF cases, PAC in response to isoproterenol was an independent predictor of AF recurrence after PVI with a cryoballoon, whereas inducibility with RAP could not predict AF recurrence.^19^ Isoproterenol for inducibility may evaluate other processes for the development of AF. Isoproterenol can detect the underlying triggers for AF, whereas RAP evaluates the substrate of atrial tachyarrhythmia remaining after the procedure. A previous study showed that the trigger for PAF is mainly localized in the PVs.^20^ RAP may reveal the remaining atrial arrhythmogenic substrate beyond the PVs. In our study, induced type of arrhythmia is often not consistent with the recurrent type of arrhythmia. Even though the inducibility by RAP may provide information on the risk of recurrence in patients with PeAF, the optimal treatment strategy including additional ablation for inducible patients is still unclear.

### 4.2 Pacing CI and inducibility

In our study, RAP was performed at the end of the catheter ablation, from 300 ms with a subsequent decrement of 10 ms or failure to capture the 1:1 atrial tissue. To the best of our knowledge, no study tested the method of inducibility protocol. Shorter pacing cycle length may be more likely to induce AF, because pacing stimulation is likely to occur during repolarization of the left atrial muscle.

Overall, the results of our study support the finding that noninducibility in shorter pacing cycle length was associated with the absence of atrial tachyarrhythmia recurrence after the procedure, especially in PeAF. Our study indicated that additional ablation and careful follow‐up after catheter ablation may be considered based on the response to the atrial pacing maneuver with short CI.

## 5 STUDY LIMITATIONS

Our study had some limitations. First, this study was a single‐center, retrospective analysis. The atrial effective refractory period was not confirmed in all cases. Moreover, the minimum pacing cycle length and additional ablation procedures depended on the operators’ direction. Second, the median follow‐up period for the patients is longer in our study than in previous observational studies. However, the postablation follow‐up period and method were different, and some patients have a short follow‐up period. Third, the voltage map was not recorded in all cases. The existence of low voltage areas is related to the high inducibility of the atrial tachyarrhythmias after the procedure.^21^ The voltage map might be useful for selecting tailored substrate‐based catheter ablation. Fourth, all our patients received catheter ablation under deep sedation. Inducibility was evaluated under conscious sedation in most of prior studies, and difference in sedation strategies may affect inducibility. Fifth, even though only 17 patients (5.7%) received rapid pacing from the right atrium, and there was no significant difference in the distribution ratio of pacing site between three groups (*P* = .305), the differences in pacing site can theoretically affect inducibility, and might have influenced the results of our study.

## 6 CONCLUSIONS

Noninducibility with a shorter cycle length predicted recurrence after catheter ablation for PeAF, whereas inducibility had only a neutral value in patients with PAF. Pacing CI and type of AF may influence the predictive value of atrial tachyarrhythmia recurrence.

## CONFLICT OF INTEREST

The authors declare no conflict of interest for this article.
